# Mathematical Equations to Predict Positive Airway Pressures for Obstructive Sleep Apnea: A Systematic Review

**DOI:** 10.1155/2015/293868

**Published:** 2015-07-30

**Authors:** Macario Camacho, Muhammad Riaz, Armin Tahoori, Victor Certal, Clete A. Kushida

**Affiliations:** ^1^Division of Otolaryngology, Sleep Surgery, and Sleep Medicine, Tripler Army Medical Center, 1 Jarrett White Road, Tripler AMC, HI 96859, USA; ^2^Sleep Medicine Division, Department of Psychiatry and Behavioral Sciences, Stanford Hospital and Clinics, Redwood City, CA 94063, USA; ^3^Sleep Disorders Center, University of Michigan, Ann Arbor, MI 48109, USA; ^4^Department of Internal Medicine, Highland Hospital, Alameda Health System, Oakland, CA 94602, USA; ^5^Department of Otorhinolaryngology/Sleep Medicine Centre, Hospital CUF, 4100-180 Porto, Portugal; ^6^CINTESIS-Centre for Research in Health Technologies and Information Systems, University of Porto, 4200-450 Porto, Portugal

## Abstract

*Objective*. To systematically review the international literature for mathematical equations used to predict effective pressures for positive airway pressure (PAP) devices.* Methods*. Google Scholar, PubMed, Scopus, Embase, Web of Science, CINAHL, and The Cochrane Library were searched through June 27, 2015. The PRISMA statement was followed. There was no language limitation.* Results*. 709 articles were screened, fifty were downloaded, and twenty-six studies presented equations that met the inclusion and exclusion criteria. In total, there were 4,436 patients in the development phases and 3,489 patients in the validation phases. Studies performed multiple linear regressions analyses as part of the equation(s) development and included the following variables: physical characteristics, polysomnography data, behavioral characteristics, and miscellaneous characteristics, which were all predictive to a variable extent. Of the published variables, body mass index (BMI) and mean oxygen saturation are the most heavily weighted, while BMI (eighteen studies), apnea-hypopnea index (seventeen studies), and neck circumference (eleven studies) were the variables most frequently used in the mathematical equations. Ten studies were from Asian countries and sixteen were from non-Asian countries.* Conclusion*. This systematic review identified twenty-six unique studies reporting mathematical equations which are summarized. Overall, BMI and mean oxygen saturation are the most heavily weighted.

## 1. Introduction

Obstructive sleep apnea (OSA) is a common health problem, with an estimated prevalence of 9% in women and 24% in men (middle-aged Americans) [1]. Although there are many medical [2–4] and surgical treatment options [5–8], positive airway pressure (PAP) therapies (i.e., automatic positive airway pressure (APAP), bilevel positive airway pressure (BPAP), or continuous positive airway pressure (CPAP)) are highly efficacious treatments for OSA [9]. Manual, attended in-laboratory PAP titration (PAP titration) studies are considered the gold standard method for prescribing PAP pressures for OSA patients [10]. PAP titration studies enable providers to prescribe a fixed pressure or a range of pressures to treat OSA whereas APAP devices utilize built-in algorithms for providing PAP pressure therapy without the requirement for a PAP titration. APAP devices may be a cost-effective alternative to PAP titration studies in carefully selected OSA patients after a focused evaluation for clinical risk factors. Various studies have assessed the utility of APAP; however, its generalizability has been questioned in terms of cardiovascular benefits with only marginal benefit in terms of PAP adherence and daytime sleepiness [11, 12]. Additionally, titration studies can be costly, and methods for increasing success have been explored such as using mathematical equations that can be used to predict effective CPAP pressures.

Researchers have used multiple linear regressions analyses on variables such as age, apnea-hypopnea index (AHI), apnea index, body mass index (BMI), craniofacial/cephalometric features, height, lowest oxygen saturation, mean oxygen saturation, neck circumference (NC), oropharyngeal soft tissues, oxygen desaturation index (ODI), race, respiratory disturbance index (RDI), sex (male versus female), sleepiness, smoking, and weight [13–52]. These multiple linear regressions analyses have been used to formulate predictive mathematical equations as compared to the effective CPAP pressures determined during PAP titration studies. The equations have subsequently been used either for estimating starting pressures or for use during in-lab PAP titration studies. With increasing healthcare costs, it is currently difficult to perform PAP titrations in every OSA patient. The objective of this study was to systematically review the international literature for mathematical equations used to predict effective positive airway pressure device pressures and to provide a summary.

## 2. Methods

As a systematic review of the currently published literature, this study is exempt from Institutional Review Board (IRB) protocol review. Authors Macario Camacho and Armin Tahoori searched Google Scholar, PubMed, Scopus, Embase, Web of Science, and The Cochrane Library from inception through January 1, 2015, initially, followed by an update through June 27, 2015. Both authors agreed on the articles for inclusion based on the predetermined inclusion and exclusion criteria and if there was a difference, it was resolved by the consensus.

### 2.1. Study Selection

Inclusion criteria were as follows: (1) OSA patients were included, (2) physical, behavioral, miscellaneous, and/or polysomnography variables were evaluated with step-wise, multiple linear regression analyses in order to develop predictive mathematical equations for determining pressure(s) for PAP devices, (3) all languages were included, and (4) there was no publication date limitation. Exclusion criteria were as follows: (1) predictive mathematical equations were not presented or (2) other techniques were used to determine PAP pressures. If additional information was needed from authors, then the plan was to contact the corresponding author for the study at least twice.

The search strategies were tailored to the specific databases and were conducted by combining MeSH terms, keywords, and phrases which would yield potential studies with predictive mathematical equations. An example search strategy for PubMed is ((“Positive-Pressure Respiration” [Mesh]) AND ((sleep [All Fields]) OR (apnea [All Fields]) OR (“Sleep Apnea Syndromes” [Mesh])) AND (predict^*∗*^ [All Fields] OR equation^*∗*^ [All Fields] OR math^*∗*^ [All Fields] OR formul^*∗*^ [All Fields] OR calculate^*∗*^ [All Fields])).

Data from each of the individual studies were cataloged. Variables collected from each study included the country performing the research, whether the study was a development and/or validation study, the number of patients, the mean ages, the mean body mass index values, the polysomnographic variables, the mathematical equations, and the accuracy of each formula. For the individual mathematical equations, each of the variables was weighted (i.e., neck circumference, apnea-hypopnea index, body mass index, etc.) differently, and the multiple linear regressions analyses determined the weight for the coefficient used to multiply each variable by in the mathematical equations, which this paper refers to as the “coefficient.” For example, in the formula 6.2 × (BMI × 0.11), the coefficient is 0.11. The coefficient was cataloged in table form, thereby providing overall ranges and a combined mean value for each variable (i.e., AHI, RDI, and BMI), thus allowing for comparison of variables between studies.

The Preferred Reporting Items for Systematic Reviews and Meta-Analyses (PRISMA) statement [53] was adhered to during this review. Given that this systematic review is not a meta-analysis, there was no statistical evaluation of the presented predictive equations. Publication bias was not assessed given the unavailability of quantitative data for meta-analysis. The National Institute for Health and Clinical Excellence (NICE) quality assessment tool for case series was utilized for study assessment [54].

## 3. Results

### 3.1. Methodological Study Quality

The quality of each study was assessed with the NICE quality assessment tool, which evaluates 8 items. The included studies were case series studies, either retrospective or prospective. Most studies satisfied between 3 and 6 of the 8 evaluated items. None of the studies were multi-institutional; see Table 1.

### 3.2. Search Results

**Figure 1:**
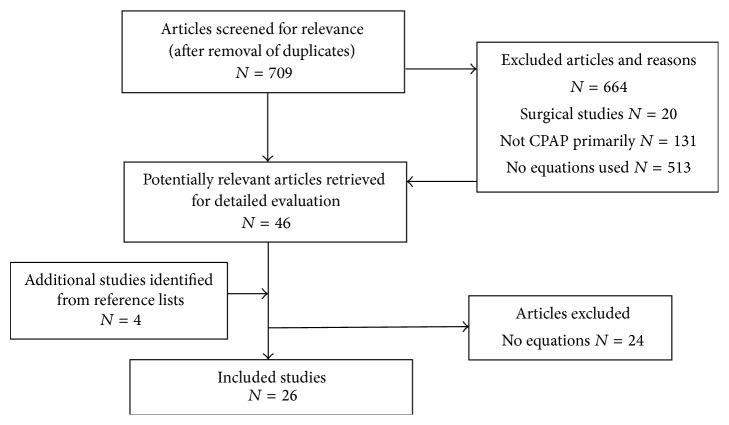
Flow diagram for studies reporting mathematical equations for predicting positive airway pressures. *N* = number of articles.

The searches yielded a total of 709 studies (after exclusion of duplicates). After screening the studies, forty-six of them were potentially relevant and the full-text versions were downloaded for detailed evaluation [13–52, 55–60] and four studies were identified after reading the references and they were also downloaded [60–64]. After detailed review of the fifty studies, the authors came to a consensus for twenty-six studies [13–15, 19, 20, 31, 32, 34, 35, 38, 43–45, 47–51, 55–61, 63, 64] which presented equations that met the inclusion and exclusion criteria (see Figure 1). There were 4,436 patients in the development phases and 3,489 patients in the validation phases for the combined studies included in this review. The earliest published study was by Miljeteig and Hoffstein [38] in 1993 and the most recent study was published in 2015 [56]. Table 1 presents the study quality assessment for the individual studies. Studies performed multiple linear regressions analyses as part of the development including physical characteristics (e.g., body mass index and neck circumferences), polysomnography (e.g., apnea-hypopnea index, respiratory disturbance index, oxygen desaturation index, lowest oxygen saturation, and mean oxygen saturation), behavioral characteristics (smoking in pack years), and miscellaneous characteristics (e.g., sleepiness and cephalometrics), which were all predictive to a variable extent. Researchers from the following countries published their predictive equations: Britain, Canada, China, Germany, Greece, Japan, Korea, Mexico, Taiwan, Turkey, and the United States of America. The results for the study authors, years, countries, ages, BMI, AHI, accuracy of the formula, and the multiple linear regressions analysis equations are presented in Table 2 (non-Asian countries) and Table 3 (Asian countries).

#### 3.2.1. Variables in the Mathematical Equations

*Body Mass Index and Ideal Body Weight*. Eighteen studies [13, 19, 20, 31, 32, 35, 38, 43–45, 47, 50, 57, 59–61, 63] included BMI and one study included percent of the ideal body weight [65] as variables in the mathematical equations. For the Asian studies, the means for BMI were between 25.1 and 28.4 kg/m^2^, while in the non-Asian studies the means for BMI were between 30.9 and 40.6 kg/m^2^. The coefficient mean value for BMI was 0.12847 and ranged between 0.02 and 0.205 and for IBW was 0.028. The BMI for Hoffstein and Mateika's validation study [28] had a large range from a low of 23 and a high of 48 kg/m^2^. The studies by Choi et al. [19] and Rowley et al. [43] also utilized BMI but the mathematical equation was not as accurate for prescribing CPAP.

*Apnea-Hypopnea Index and Respiratory Disturbance Index*. AHI was a variable in mathematical equations for seventeen studies [13, 20, 32, 35, 38, 43–45, 56–60, 64, 66] and RDI for four studies [31, 34, 55, 65]. For all patients combined, the coefficient mean value for AHI was 0.0442 and ranged between 0.01 and 0.18. For the Asian studies the mean for AHI ranged between 33.9 and 58 events/hr, while in the non-Asian studies the mean for AHI ranged between 30 and 56.7 events/hr. The coefficient mean value for RDI was 0.02475 and ranged between 0.01301 and 0.041. Rowley et al.'s study [43] increased the success rate of titration protocols from 50 to 68% (AHI decreased by 50% and a final AHI ≤10 events/hr at final tested pressure). Seven studies used the mathematical equation in order to derive a starting PAP titration pressure; thereby the technicians would start at a pressure that was closer to the actual therapeutic pressure [15, 35, 38, 45, 48, 58, 59].

*Oxygen Desaturation Index, Mean, and Lowest Oxygen Saturation*. Five studies included oxygen desaturation index [15, 20, 48, 50, 64], four studies evaluated mean oxygen saturation [13, 14, 34, 50], and four studies evaluated lowest oxygen saturation [31, 34, 57, 65] as variables in the mathematical equations. The coefficient mean value for ODI was 0.04417 and ranged between 0.01 and 0.133. For the mean oxygen saturation the overall coefficient mean value was 0.0441 and ranged between 0.06 and 0.312. The coefficient mean value for the lowest oxygen saturation was 0.065 and ranged between 0.05 and 0.071. Effective pressure in Basoglu and Tasbakan's study [15] was more significantly correlated to ODI than BMI and AHI, and Stradling et al. [48] combined neck circumference with ODI (≥4% oxygen desaturation/hr). Other studies that used ODI but not neck circumference had poor accuracy (e.g., Torre-Bouscoulet et al. [50, 51] who also utilized BMI and ODI).

*Cephalometric Variables*. Cephalometric variables included in the mathematical equations by Ito et al. (tongue area and lower face cage ratio) [66], Akahoshi et al. (the angle between a line from point B to the menton and from the menton to the hyoid bone, coefficient of 0.041) [13], and Akashiba et al. (cranial base flexure, coefficient of 0.099) [14].

*Sex and Race*. Male versus female sexes were factored into the mathematical equations of three studies [44, 51, 60]. For race, ten studies were from Asian countries and sixteen studies from non-Asian countries. No significant difference was found between the predicted pressures when Basoglu and Tasbakan [15] compared their predictive mathematical equation with Hoffstein and Mateika's mathematical equation [28] derived from a Caucasian population and Lin et al.'s mathematical equation [32] derived from an Asian population. Additionally, Basoglu and Tasbakan [15] found that their equation was significantly correlated with both of these equations.

*Additional Variables*. The updated Friedman Tongue Position was a variable in the study by Lai et al. [56]. Smoking in pack years was a variable in the formulas derived by Schiza et al. [44]. The snoring severity score was incorporated into the mathematical equation by Anees et al. [63]. The Epworth Sleepiness Scale Score was a variable in the study by Lee et al. [31]. When comparing the eleven studies in our review that utilized neck circumference for derivation of the mathematical equations [15, 28, 35, 38, 43, 45, 48, 58–61], seven of these successfully predicted therapeutic PAP. The coefficient for neck circumference ranged between 0.01 and 0.16.

## 4. Discussion

There are four main findings in this systematic review. First, body mass index was the most common variable in the mathematical equations, being included in eighteen studies [13, 19, 20, 31, 32, 35, 38, 43–45, 47, 50, 57, 59–61, 63], and one study also included percent of the ideal body weight [65]. The coefficient mean value for BMI was 0.12847 and ranged between 0.02 and 0.205 and for IBW was 0.028. As BMI increases, excessive fat deposits around the neck and within the pharynx and this can affect the collapsibility of upper airway [67]. The BMI for Hoffstein and Mateika's validation study [28] had a large range from a low of 23 kg/m^2^ and a high of 48 kg/m^2^. The studies by Choi et al. [19] and Rowley et al. [43] also utilized BMI but the mathematical equation was not as accurate for prescribing CPAP. In the Asian studies, the mean value for the coefficient was 0.16871, while, in the non-Asian studies, the mean value for the coefficient was 0.1003, demonstrating that this variable emerged as a more heavily weighted variable than other variables during the multiple linear regressions analyses. Given that for the Asian studies, the means for BMI were between 25.1 kg/m^2^ and 28.4 kg/m^2^, while in the non-Asian studies the means for BMI were between 30.9 kg/m^2^ and 40.6 kg/m^2^; therefore, it is possible that smaller differences in BMI for patients who are overweight (but who are not obese) can make a larger difference in deriving a therapeutic treatment pressure. It is logical to reason that as patients gain weight and increase their body mass index a higher amount of positive airway pressure would be needed to overcome the additional upper airway resistance as well as the abdominal mass pushing against the diaphragm.

Second, polysomnographic variables were also important during the multiple linear regressions analyses. Apnea-hypopnea index was a variable in mathematical equations for seventeen studies [13, 20, 32, 35, 38, 43–45, 56–60, 64, 66] and RDI for four studies [31, 34, 55, 65]. The coefficient for AHI ranged between 0.01 and 0.18 and for RDI ranged between 0.01301 and 0.041. The mean overall coefficient for AHI was 0.0442 for all studies and 0.03963 for Asian and 0.04878 for non-Asian studies. In the mathematical equation by Loredo et al. [34] RDI was a variable in the mathematical equation and there was overall good prediction which demonstrates that respiratory effort related arousals (RERAs) could be a possible factor that may influence the derived mathematical equations. For the five studies that included oxygen desaturation index [15, 20, 48, 50, 64], four studies that evaluated mean oxygen saturation [13, 14, 34, 50], and the four studies that evaluated lowest oxygen saturation [31, 34, 57, 65], the factor that had the largest influence when used was the mean oxygen saturation. The mean coefficient value for mean oxygen saturation was 0.19525, compared to 0.04417 for oxygen desaturation index and 0.065 for lowest oxygen saturation. Given the fact that the lowest oxygen saturation generally lasts seconds per event, it makes sense that the mean oxygen saturation would end up having more weight in the formulas given that mean oxygen saturation takes into account the oxygen saturation throughout the entire night. Equations that include ODI as a variable might be helpful in instances where patients have a high likelihood of underlying oxygen desaturation during sleep. The study by Akashiba et al. [14] reported a correlation between the mean oxygen saturation during sleep and optimal CPAP pressure, demonstrating that a large percentage of OSA patients in the study had hypercapnia and underlying obesity hypoventilation syndrome which is a 24-hour condition, and this fact can confound the results.

Third, race/ethnicity and gender may affect PAP pressures. The study by Lin et al. [32] demonstrated that race/ethnicity appear to influence the PAP pressures needed to treat OSA; however, the results are variable across studies. Basoglu and Tasbakan's [15] mathematical equation correlated to both a Caucasian and an Asian mathematical equation and this may be in part due to the fact that Turkey is composed of mixed racial/ethnic groups which include patients from both European and Asian descent. In contrast, Lin et al.'s mathematical equation was only valid for Taiwanese patients [32]. Given that the mean value for the BMI coefficient was 0.16781 for Asian studies and 0.1003 for non-Asian studies, it is possible that the reason that race/ethnicity influences PAP pressures has more to do with the BMI since the patients in the non-Asian studies have a significantly higher BMI, which affects the derivation of the mathematical equation(s). Gender also contributes to the ideal predicted pressures as demonstrated by the different equations derived for men versus women, as shown in studies by Schiza et al. [44], Torre-Bouscoulet et al. [50, 51], and Teschler et al. [60] Factors affecting the predictive pressures for PAP between males and females include that males generally have a higher AHI and a longer soft palate [46, 68]. In the three studies reporting separate mathematical equations for men versus women, the coefficient was higher for men, so that even if all the other variables are the same, the final mathematical equation derived pressures are higher for men than women.

Fourth, additional research is needed, with three topics receiving priority. First, given that the quality of sleep, as well as density and duration of rapid eye movement (REM) sleep, can be highly variable among OSA patients, it is possible that REM sleep differences among sleep apnea patients can affect the pressure needs irrespective of the severity. Studies evaluating the differences in required pressures based on sleep stage would help determine these differences. A patient who has a significant proportion of REM sleep may end up needing a higher amount of pressure secondary to the upper airway relaxation. Thus far, only Tofts et al. [58] have evaluated the effect of AHI during REM; however, the AHI during NREM was not evaluated as a separate variable; rather the AHI in its entirety was included in the formula. A second area of research is whether the pressure requirements are really correlated with apnea-hypopnea index as a whole, or it may be that apneas and hypopneas should have different coefficients as it is logical that an apnea would require higher pressures than a hypopnea to maintain a patent airway. Only, Tofts et al. [58] have evaluated the effect of hypopnea as a variable, but the apneas were not included as variable in their mathematical equation. The contribution that hypopneas versus apneas can make is difficult to answer, as two different patients with the same AHI but a different proportion of apneas and hypopneas might require different pressures to overcome these obstructive events, thus potentially making mathematical equations that do not separate the two variables less accurate. Third, although the mathematical equations have helped improve PAP titration study success, the formulas are not completely generalizable secondary to physical, behavioral, comorbidity, and polysomnography differences in OSA patients. More studies evaluating the utility of mathematical equations for prescribing CPAP for home use are needed.

*Limitations*. Despite the best effort of the two searching authors (Macario Camacho and Armin Tahoori) to identify all the currently published predictive mathematical equations in a systematic fashion, it is possible that we failed to identify one or more equations. It is possible that studies demonstrating no utility or benefit from using a predictive equation never made it to publication secondary to publication bias against negative studies; therefore, they did not make it into this review. However, given that there were studies that demonstrated poor accuracy, at least some of these studies made it to publication despite this fact. Another limitation is that most studies did not report the head and neck examination findings to include the nasal examination (i.e., grading the size of the inferior turbinates [69]), as it has been demonstrated that head and neck anatomy and surgery correcting obstruction (specifically, nasal obstruction) can lower the required pressures by 2-3 centimeters of water pressure and also increased CPAP use in a currently published meta-analysis [70].

## 5. Conclusion

This systematic review identified twenty-six unique studies reporting mathematical equations which are summarized in this review. Overall, body mass index and mean oxygen saturation are the most heavily weighted of all the published variables.

## Figures and Tables

**Table 1:** Quality assessment of the included studies checklist from questions from National Institute for Health and Clinical Excellence (NICE) 1–8: (1) Is case series collected in more than one center? (2) Is the hypothesis/aim/objective of the study clearly described? (3) Are the inclusion and exclusion criteria clearly reported? (4) Is there a clear definition of the outcomes reported? (5) Were data collected prospectively? (6) Is there an explicit statement that patients were recruited consecutively? (7) Are the main findings of the study clearly described? (8) Are outcomes stratified? Abbreviations: ABS = abstract; NA = not applicable (abstract); PCS = prospective case series; RCS = retrospective case series.

Authors	Study type	Study design	Year	Location	Quality assessment of included studies							
					1	2	3	4	5	6	7	8
Lai et al. [56]	RCS	Development	2015	Taiwan	No	Yes	No	Yes	No	No	Yes	Yes

Ito et al. [66]	RCS	Development	2014	Japan	No	Yes	Yes	Yes	No	No	Yes	Yes

Wu et al. [57]	PCS	Development Validation	2014	Taiwan	No	Yes	No	Yes	Yes	Yes	Yes	Yes

Luo et al. [35]	RCS	Development Validation	2013	China	No	Yes	Yes	Yes	No	Yes	Yes	Yes

Lee et al. [31]	RCS	Development Validation	2013	Korea	No	Yes	Yes	No	No	Yes	Yes	Yes

Basoglu and Tasbakan [15]	RCS	Development Validation	2012	Turkey	No	Yes	Yes	Yes	Yes	No	Yes	Yes

Tofts et al. [58]	RCS	Development Validation	2012	USA	NA	NA	NA	NA	NA	NA	NA	NA

Schiza et al. [44]	RCS	Development Validation	2011	Greece	No	Yes	Yes	Yes	No	No	Yes	No

Anees [63]	PCS	Validation	2010	USA	NA	NA	NA	NA	NA	NA	NA	NA

Choi et al. [19]	RCS	Development Validation	2010	Korea	No	Yes	No	Yes	No	Yes	Yes	Yes

Akahoshi et al. [13]	PCS	Development Validation	2009	Japan	No	Yes	No	Yes	Yes	Yes	Yes	Yes

Chuang et al. [20]	PCS	Development Validation	2008	Taiwan	No	Yes	No	Yes	Yes	No	Yes	No

El Solh et al. [59]	RCS	Development Validation	2007	USA	No	Yes	No	Yes	No	Yes	Yes	Yes

Loredo et al. [34]	PCS	Development Validation	2007	USA	No	Yes	Yes	Yes	Yes	Yes	Yes	No

Skomro et al. [47]	RCS	Development Validation	2007	Canada	No	Yes	Yes	Yes	No	No	Yes	No

Torre-Bouscoulet et al. [50, 51]	RCS	Development Validation	2007 2009	Mexico	No	Yes	Yes	No	No	No	Yes	No

Panagou et al. [64]	ABS	Development	2005	Greece	NA	NA	NA	NA	NA	NA	NA	NA

Rowley et al. [43]	RCS	Development Validation	2005	USA	No	Yes	No	Yes	No	No	Yes	Yes

Stradling et al. [48, 49]	RCS	Development Validation	2004	Britain	No	Yes	No	Yes	Yes	No	Yes	Yes

Lin et al. [32]	PCS	Development Validation	2003	Taiwan	No	Yes	Yes	Yes	Yes	Yes	Yes	Yes

Akashiba et al. [14]	PCS	Development Validation	2001	Japan	No	Yes	No	Yes	Yes	No	Yes	No

Sériès [45]	PCS	Development Validation	2000	Canada	No	Yes	No	Yes	Yes	Yes	Yes	Yes

Nahmias et al. [65]	ABS	Development	1995	USA	NA	NA	NA	NA	NA	NA	NA	NA

Teschler et al. [60]	PCS	Development Validation	1995	Germany	No	Yes	No	Yes	Yes	No	Yes	Yes

Hoheisel and Teschler [61]	ABS	Development	1994	Germany	NA	NA	NA	NA	NA	NA	NA	NA

Miljeteig and Hoffstein [38]	PCS	Development Validation	1993	Canada	No	Yes	No	Yes	Yes	No	Yes	Yes

**Table 2:** Study demographics and mathematical equations for studies from non-Asian countries. Definitions: %IBW = percent of ideal body weight, AHI = apnea-hypopnea index, BMI = body mass index (in kg/m^2^), CPAP = continuous positive airway pressure, cwp = centimeters of water pressure, ESS = Epworth Sleepiness Scale [71], HI = health index, Msat = mean saturation, nadir SaO_2_ = lowest oxygen saturation, NC = neck circumference, NR = not reported, Nsat = nadir saturation, ODI = oxygen desaturation index, PAP = positive airway pressure, Peff = effective pressure, Ppred = predicted pressure, pts = patients; RDI = respiratory disturbance index, spO_2_ = oxygen saturation, SSS = snoring severity score, USA = United States of America, and *C*, *X*, *Y*, and *Z* = coefficients based on artificial neural networks.

Study group, year, and country	Number of pts Development (D) Validation (V)	Mean age	Mean BMI	Mean AHI	Mathematical equation	Accuracy of formula
Basoglu and Tasbakan (2012), Turkey [15]	D = 250 V = 130	52.3 ± 10.3	32.3 ± 5.3	56.7 ± 22.8	0.148 × NC + (0.038 × ODI)	Within ±3 cwp in 96.2% of pts

Tofts et al. (2012), USA [58]	D/V = 310	NR	NR	NR	5.55 + 0.05327 (HI) + 0.03276 (NC) + 0.03422 (AHI Crude) + 0.0005568 (AHI Supine) + 0.001110 (AHI REM) + 0.01301 (RDI)	Predicts 30% of the variability while being within ±2 cwp 74% of the time

Schiza et al. (2011), Greece [44]	D/V = 1111	54.6 ± 10.67	34.35 ± 6.03	41.5 ± 20.16	Men: 5.16 + (0.003 × smoking in pack years) + (0.054 × BMI) + (0.016 × AHI) − 0.403 Women: 5.16 + (0.003 × smoking in pack years) + (0.054 × BMI) + (0.016 × AHI) − 0.806	Within ±2 cwp of the effective pressure in 95% of pts

Anees (2010), USA [63]	V = 27	—	—	—	0.086 × BMI + 0.029 × SSS + 5.989	Within ±2 cwp of CPAP titration in 44%; 2 cwp higher than predicted in 37%

El Solh et al. (2007), USA [59]	D = 311 V = 98	49.6 ± 12.4	35 (34–37)	33 (28–38)	*X* − NC + *Y* − BMI + *Z* × AHI + *C*	Correlation coefficients between the titration study and predicted pressure was 0.86. The equation underestimated optimal pressures

Loredo et al. (2007), USA [34]	D/V = 76	47.6 ± 9.8	31.3 ± 5.4	55.5 ± 31.3 RDI	30.8 + 0.03 × RDI − 0.05 × NSAT − 0.2 × MSAT	Equation predicted 67% of the variance for Peff

Skomro et al. (2007), Canada [47]	D/V = 183	51 ± 11	37 ± 8	46 ± 33	6.2 × [BMI × 0.11]	Empiric CPAP pressure was suboptimal in 40% pts

Torre-Bouscoulet et al. (2007/2009), Mexico [50, 51]	D/V = 100	49 ± 11	34 ± 4	≥30	Men: (BMI × 0.09) + (ODI × 0.01) − (mean SpO_2_ × 0.06) + 11.9 Women: (BMI × 0.07) + (ESS × 0.1) + (ODI × 0.02) + 5.33	Poor agreement between 95% APAP pressures and predictive equations. Equations were not successful

Panagou et al. (2005), Greece [64]	D = 26	51 ± 11.2	—	—	4.95 + (0.18 × AHI) − (0.133 × DI)	No validation testing

Stradling et al. (2004), Britain [48, 49]	D = 101 V = 30	49.0 ± 10.5	36.5 ± 6.5		(0.048 × 4% saO_2_ dips/h) + (0.128 × NC) + 2.1	Considerable PAP variations from night to night. Similar results for APAP trial and CPAP titration

Rowley et al. (2005), USA [43]	D = 224 V = 192	50.5 ± 9.5	40.6 ± 8.8	32.0 ± 26.4	(0.16 × BMI) + (0.13 × NC) + (0.04 × AHI) − 5.12	Equation improves success rate of in lab titrations but equations were not as helpful for prescribing CPAP

Sériès (2000), Canada [45]	D/V = 40	—	—	46.1 ± 26.3	0.193 × BMI + 0.077 × NC + 0.02 × AHI − 0.611	Home APAP titration successfully predict fixed CPAP in 95% of pts

Nahmias et al. (1995) [65]	D/V = 40	—	—	37.7 ± 3.3	8.7 + 0.028 × %IBW + 0.015 × RDI − 0.071 × nadir SaO_2_	In 73% of patients, the equation predicted CPAP ≥ CPAP titration study

Teschler et al. (1995), Germany [60]	D = 77 V = 180	55 ± 10	30.9 ± 5.8	38 ± 21	1.95 + 0.80 × sex factor (men = 2, women = 1) + 0.09 × BMI + 0.01 × NC + 0.03 × AHI	Mean CPAP pressure was 9.1 ± 2.0 cwp and the predicted was 8.4 ± 3.6 cwp. In 51% of pts the difference was greater than ±1 cwp

Hoheisel and Teschler (1994), Germany [61]	—	—	—	—	(0.07 × NC) + (0.02 × BMI) + (0.03 × AHI) + 3.2	Calculations were made to attempt to improve home prescriptions

Miljeteig and Hoffstein (1993), Canada [38]	D = 208 V = 129	50 ± 11	34 ± 8	50 ± 31	−5.12 + 0.13 × BMI + 0.16 × NC + 0.04 × AHI	Within ±2 cwp in 75% of pts. The equation accounted for 76% of the variability in CPAP

**Table 3:** Study demographics and mathematical equations from Asian countries. Definitions: AHI = apnea-hypopnea index, APAP = automatic positive airway pressure, BMeH = the angle between a line from point B to the menton and from the menton to the hyoid bone, BMI = body mass index (in kg/m^2^), CPAP = continuous positive airway pressure, cwp = centimeters of water pressure, DI = desaturation index, ESS = Epworth Sleepiness Scale [71], HMD = hyoid-mental distance, LFC = lower face cage, NC = neck circumference, NSBa = cranial base flexure (cephalometrics), PAP = positive airway pressure, Peff = effective pressure, PnCPAP = optimal nasal continuous positive airway pressure, RDI = respiratory disturbance index, saO_2_ = saturation of oxygen, spO_2_ = oxygen saturation, TG = tongue area, and uFTP = updated Friedman's tongue position.

Study group, year, and country	Number of pts Development (D) Validation (V)	Mean age	Mean BMI	Mean AHI	Mathematical equation	Accuracy of formula
Lai et al. (2015), Taiwan [56]	D = 129	46.2 ± 11.0	27.1 ± 3.6	43.3 ± 22.5	1.01 uFTP + 0.74 HMD + 0.059 AHI − 1.603	No prospectively validation testing performed

Ito et al. (2014), Japan [66]	D = 66 V = 66	—	25.1 (21.2, 30.4)	33.9 (19.5, 59.9)	1.000 + 0.043 × AHI + 9.699 × TG/LFC	Equation accounted for 28% of the total variance in PnCPAP

Wu et al. (2014), Taiwan [57]	D = 57 V = 30	53.3 ± 13.1	28.1 ± 3.5	53.6 ± 18.3	6.380 + 0.033 × AHI − 0.068 × SaO_2_ nadir + 0.171 × BMI	Within ±1 cwp in 30%, within ±2 cwp in 56.7%, and within ±3 cwp in 86.7% of validation pts

Luo et al. (2013), China [35]	D = 51	48.0 ± 11.3	28.0 ± 4.1	54.3 ± 18.9	0.05 × AHI + 0.15 × BMI + 0.066 × NC − 1.712	*R* = 0.677, *R* ^2^ = 0.459; calculated pressure 7.7 ± 1.4 cwp versus 7.3 ± 1.5 cwp. CPAP titration was compared to APAP, and APAP pressure was higher

Lee et al. (2013), Republic of Korea [31]	Group 1: D = 178 Group 2: V = 178	51.7 ± 10.6	26.3 ± 3.6	40.1 ± 29.0	6.656 × 0.156 × BMI − 0.071 × minimal spO_2_% + 0.041 × RDI + 0.094 × ESS	Equation accounted for 38.9% of the total variance. Predicted the titration CPAP pressure in 38.8%

Choi et al. (2010), Republic of Korea [19]	D/V = 202	44.8 ± 8.5	27.6 ± 3.4	36.6 ± 25.1	0.681 + (0.205 × BMI) + (0.040 × AHI)	Equation accounted for 42% variance for optimal CPAP

Akahoshi et al. (2009), Japan [13]	D = 170 V = 110	52.9 ± 12.4	27.8 ± 4.7	50.1 ± 18.8	27.78 + (0.041 × BMeH) + (0.141 × BMI) + (0.040 × AHI) − (0.312 × mean SaO_2_)	Optimal CPAP (9.5 ± 3.0 and 9.2 ± 2.1 cwp) similar to calculated pressure respectively. Equation accounted for 47% of variance

Chuang et al. (2008), Taiwan [20]	D = 418 V = 124	49 ± 12	28.4 ± 4.4	58 ± 23	1.98 + 0.184 × BMI + 0.01 × AHI + 0.016 × DI	Successful prediction ±2 cwp of the effective pressure in 84% of study group and 73% in validated group. Equation accounted for 28% of the total variance

Lin et al. (2003), Taiwan [32]	D/V = 121	49.2 ± 12.6	28.3 ± 4.0	53.8 ± 23.6	0.52 + 0.174 × BMI + 0.042 × AHI	Successful prediction ±2 cwp of the effective pressure in 86% in validation patients

Akashiba et al. (2001), Japan [14]	D/V = 27	51.5 ± 9.6	28.1 ± 2.7	54.7 ± 22.6	42.036 − 0.209 × mean SaO_2_ − 0.099 × NSBa	Equation accounts for 57.5% of the total variance

## References

[1] Young T., Palta M., Dempsey J., Skatrud J., Weber S., Badr S. (1993). The occurrence of sleep-disordered breathing among middle-aged adults. *The New England Journal of Medicine*.

[2] Camacho M., Certal V., Abdullatif J. (2015). Myofunctional therapy to treat obstructive sleep apnea: a systematic review and meta-analysis. *Sleep*.

[3] Kumar A. R., Guilleminault C., Certal V., Li D., Capasso R., Camacho M. (2015). Nasopharyngeal airway stenting devices for obstructive sleep apnoea: a systematic review and meta-analysis. *The Journal of Laryngology and Otology*.

[4] Main C., Liu Z., Welch K., Weiner G., Jones S. Q., Stein K. (2009). Surgical procedures and non-surgical devices for the management of non-apnoeic snoring: a systematic review of clinical effects and associated treatment costs. *Health Technology Assessment*.

[5] Camacho M., Certal V., Capasso R. (2013). Comprehensive review of surgeries for obstructive sleep apnea syndrome. *Brazilian Journal of Otorhinolaryngology*.

[6] Certal V. F., Zaghi S., Riaz M. (2015). Hypoglossal nerve stimulation in the treatment of obstructive sleep apnea: a systematic review and meta-analysis. *The Laryngoscope*.

[7] Camacho M., Certal V., Brietzke S. E., Holty J.-E. C., Guilleminault C., Capasso R. (2014). Tracheostomy as treatment for adult obstructive sleep apnea: a systematic review and meta-analysis. *The Laryngoscope*.

[8] Camacho M., Liu S. Y., Certal V., Capasso R., Powell N. B., Riley R. W. (2015). Large maxillomandibular advancements for obstructive sleep apnea: an operative technique evolved over 30 years. *Journal of Cranio-Maxillofacial Surgery*.

[9] Sullivan C. E., Berthon-Jones M., Issa F. G., Eves L. (1981). Reversal of obstructive sleep apnoea by continuous positive airway pressure applied through the nares. *The Lancet*.

[10] Kushida C. A., Chediak A., Berry R. B. (2008). Clinical guidelines for the manual titration of positive airway pressure in patients with obstructive sleep apnea. *Journal of Clinical Sleep Medicine*.

[11] Ip S., D'Ambrosio C., Patel K. (2012). Auto-titrating versus fixed continuous positive airway pressure for the treatment of obstructive sleep apnea: a systematic review with meta-analyses. *Systematic Reviews*.

[12] Karasulu L., Epöztürk P. Ö., Sökücü S. N., Dalar L., AltIn S. (2010). Improving heart rate variability in sleep apnea patients: differences in treatment with auto-titrating positive airway pressure (APAP) versus conventional CPAP. *Lung*.

[13] Akahoshi T., Akashiba T., Kawahara S. (2009). Predicting optimal continuous positive airway pressure in Japanese patients with obstructive sleep apnoea syndrome. *Respirology*.

[14] Akashiba T., Kosaka N., Yamamoto H., Ito D., Saito O., Horie T. (2001). Optimal continuous positive airway pressure in patients with obstructive sleep apnoea: role of craniofacial structure. *Respiratory Medicine*.

[15] Basoglu O. K., Tasbakan M. S. (2012). Determination of new prediction formula for nasal continuous positive airway pressure in Turkish patients with obstructive sleep apnea syndrome. *Sleep & Breathing*.

[16] Beninati W., Sanders M. H. (2001). Optimal continuous positive airway pressure for the treatment of obstructive sleep apnea/hypopnea. *Sleep Medicine Reviews*.

[17] Berkani M., Lofaso F., Chouaid C. (1998). CPAP titration by an auto-CPAP device based on snoring detection: a clinical trial and economic considerations. *The European Respiratory Journal*.

[18] Choi J. H., Jun Y. J., Oh J. I. (2013). Optimal level of continuous positive airway pressure: auto-adjusting titration versus titration with a predictive equation. *Annals of Otology, Rhinology & Laryngology*.

[19] Choi J. H., Kim E. J., Kim K. W. (2010). Optimal continuous positive airway pressure level in Korean patients with obstructive sleep apnea syndrome. *Clinical and Experimental Otorhinolaryngology*.

[20] Chuang M.-L., Lin I.-F., Vintch J. R. E., Liao Y.-F. (2008). Predicting continuous positive airway pressure from a modified split-night protocol in moderate to severe obstructive sleep apnea-hypopnea syndrome. *Internal Medicine*.

[21] El Solh A., Akinnusi M., Patel A., Bhat A., TenBrock R. (2009). Predicting optimal CPAP by neural network reduces titration failure: a randomized study. *Sleep & Breathing*.

[22] Fitzpatrick M. F., Alloway C. E. D., Wakeford T. M., MacLean A. W., Munt P. W., Day A. G. (2003). Can patients with obstructive sleep apnea titrate their own continuous positive airway pressure?. *The American Journal of Respiratory and Critical Care Medicine*.

[23] Galetke W., Randerath W. J., Stieglitz S. (2009). Comparison of manual titration and automatic titration based on forced oscillation technique, flow and snoring in obstructive sleep apnea. *Sleep Medicine*.

[24] Gokcebay N., Iqbal S., Yang K., Zebrak A., Hirshkowitz M. (1996). Accuracy of CPAP predicted from anthropometric and polysomnographic indices. *Sleep*.

[25] Hertegonne K. B., Volna J., Portier S., De Pauw R., Van Maele G., Pevernagie D. A. (2008). Titration procedures for nasal CPAP: automatic CPAP or prediction formula?. *Sleep Medicine*.

[26] Hoekema A., Stegenga B., van der Aa J. G., Meinesz A. F., van der Hoeven J. H., Wijkstra P. J. (2006). Nap-titration: an effective alternative for continuous positive airway pressure titration. *Respiratory Medicine*.

[27] Hoffstein V. (1997). Accuracy of CPAP predicted from anthropometric and polysomnographic indices. *Sleep*.

[28] Hoffstein V., Mateika S. (1994). Predicting nasal continuous positive airway pressure. *The American Journal of Respiratory and Critical Care Medicine*.

[29] Hukins C. A. (2005). Arbitrary-pressure continuous positive airway pressure for obstructive apnea syndrome. *American Journal of Respiratory and Critical Care Medicine*.

[30] Lacedonia D., Sabato R., Carpagnano G. E. (2012). Predictive equations for CPAP titration in OSAS patients. *Sleep & Breathing*.

[31] Lee G.-H., Kim M. J., Lee E. M., Kim C. S., Lee S.-A. (2013). Prediction of optimal CPAP pressure and validation of an equation for asian patients with obstructive sleep apnea. *Respiratory Care*.

[32] Lin I.-F., Chuang M.-L., Liao Y.-F., Chen N.-H., Li H.-Y. (2003). Predicting effective continuous positive airway pressure in Taiwanese patients with obstructive sleep apnea syndrome. *Journal of the Formosan Medical Association*.

[33] Lofaso F., Lorino A. M., Duizabo D. (1996). Evaluation of an auto-nCPAP device based on snoring detection. *European Respiratory Journal*.

[34] Loredo J. S., Berry C., Nelesen R. A., Dimsdale J. E. (2007). Prediction of continuous positive airway pressure in obstructive sleep apnea. *Sleep & Breathing*.

[35] Luo J., Xiao S., Qiu Z., Song N., Luo Y. (2013). Comparison of manual versus automatic continuous positive airway pressure titration and the development of a predictive equation for therapeutic continuous positive airway pressure in Chinese patients with obstructive sleep apnoea. *Respirology*.

[36] Marrone O., Salvaggio A., Romano S., Insalaco G. (2008). Automatic titration and calculation by predictive equations for the determination of therapeutic continuous positive airway pressure for obstructive sleep apnea. *Chest*.

[37] Masa J. F., Jiménez A., Durán J. (2004). Alternative methods of titrating continuous positive airway pressure: a large multicenter study. *American Journal of Respiratory and Critical Care Medicine*.

[38] Miljeteig H., Hoffstein V. (1993). Determinants of continuous positive airway pressure level for treatment of obstructive sleep apnea. *The American Review of Respiratory Disease*.

[39] Miyata S., Noda A., Nakata S. (2007). Daytime polysomnography for early diagnosis and treatment of patients with suspected sleep-disordered breathing. *Sleep & Breathing*.

[40] Oksenberg A., Arons E., Froom P. (2006). Does the severity of obstructive sleep apnea predict patients requiring high continuous positive airway pressure?. *The Laryngoscope*.

[41] Oliver Z., Hoffstein V. (2000). Predicting effective continuous positive airway pressure. *Chest*.

[42] Rigau J., Montserrat J. M., Wöhrle H. (2006). Bench model to simulate upper airway obstruction for analyzing automatic continuous positive airway pressure devices. *Chest*.

[43] Rowley J. A., Tarbichi A. G. S., Badr M. S. (2005). The use of a predicted CPAP equation improves CPAP titration success. *Sleep & Breathing*.

[44] Schiza S. E., Bouloukaki I., Mermigkis C. (2011). Utility of formulas predicting the optimal nasal continuous positive airway pressure in a Greek population. *Sleep & Breathing*.

[45] Sériès F. (2000). Accuracy of an unattended home CPAP titration in the treatment of obstructive sleep apnea. *The American Journal of Respiratory and Critical Care Medicine*.

[46] Sforza E., Krieger J., Bacon W., Petiau C., Zamagni M., Boudewijns A. (1995). Determinants of effective continuous positive airway pressure in obstructive sleep apnea: role of respiratory effort. *American Journal of Respiratory and Critical Care Medicine*.

[47] Skomro R. P., Cotton D. J., Gjevre J. A. (2007). An empirical continuous positive airway pressure trial for suspected obstructive sleep apnea. *Canadian Respiratory Journal*.

[48] Stradling J. R., Hardinge M., Paxton J., Smith D. M. (2004). Relative accuracy of algorithm-based prescription of nasal CPAP in OSA. *Respiratory Medicine*.

[49] Stradling J. R., Hardinge M., Smith D. M. (2004). A novel, simplified approach to starting nasal CPAP therapy in OSA. *Respiratory Medicine*.

[50] Torre-Bouscoulet L., Escárcega E. L., Maldonado A. C., García J. C. V., Vargas M. S. M., Pérez-Padilla R. (2007). Continuous positive airway pressure used by adults with obstructive sleep apneas after prescription in a public referral hospital in Mexico City. *Archivos de Bronconeumologia*.

[51] Torre-Bouscoulet L., Castorena-Maldonado A., López-Escárcega E., Vázquez-García J. C., Pérez-Padilla R. (2009). Agreement between 95th percentile pressure based on a 7-night auto-adjusting positive airway pressure trial vs. equation-based predictions in sleep apnea. *Journal of Clinical Sleep Medicine*.

[52] West S. D., Jones D. R., Stradling J. R. (2006). Comparison of three ways to determine and deliver pressure during nasal CPAP therapy for obstructive sleep apnoea. *Thorax*.

[53] Moher D., Liberati A., Tetzlaff J., Altman D. G. (2009). Preferred reporting items for systematic reviews and meta-analyses: the PRISMA statement. *PLoS Medicine*.

[54] National Institute for Health and Clinical Excellence (2009). *Methods for Development of NICE Public Health Guidance*.

[55] Tofts R., Dozier J., Daco J. (2011). Predictive formulas for CPAP titration; a comparison and reassessment. *Chest*.

[56] Lai C.-C., Friedman M., Lin H.-C. (2015). Clinical predictors of effective continuous positive airway pressure in patients with obstructive sleep apnea/hypopnea syndrome. *The Laryngoscope*.

[57] Wu M.-F., Hsu J.-Y., Huang W.-C. (2014). Should sleep laboratories have their own predictive formulas for continuous positive airway pressure for patients with obstructive sleep apnea syndrome?. *Journal of the Chinese Medical Association*.

[58] Tofts R., Hernandez M., Agarwal A. (2012). Algorithm based CPAP titration formulas: a comparison and review. *Chest*.

[59] El Solh A. A., Aldik Z., Alnabhan M., Grant B. (2007). Predicting effective continuous positive airway pressure in sleep apnea using an artificial neural network. *Sleep Medicine*.

[60] Teschler H., Hoheisel G., Wagner B., Schumann H., Konietzko N. (1995). Predictive factors of minimally effective nCPAP ventilation in treatment of obstructive sleep apnea?. *Medizinische Klinik*.

[61] Hoheisel G. B., Teschler H. (1994). Clinical parameters for the presciption of minimal effective CPAP for the treatment of obstructive sleep apnea. *American Journal of Respiratory and Critical Care Medicine*.

[62] Jenkinson C., Davies R. J. O., Mullins R., Stradling J. R. (1999). Comparison of therapeutic and subtherapeutic nasal continuous positive airway pressure for obstructive sleep apnoea: a randomised prospective parallel trial. *The Lancet*.

[63] Anees S. (2010). Application of patient reported snoring severity (PRSS) to predict continuous positive airway pressure (CPAP) in patients with suspected obstructive sleep apnea (OSA) awaiting overnight polysomnography (NPSG): a prospective study. *Sleep*.

[64] Panagou P., Mermingis H., Tsipra S., Psathakis K., Tsintiris K. (2005). Prediction of effective continuous positive airway pressure (CPAP) in obstructive sleep apnea syndrome (OSAS). *The European Respiratory Journal*.

[65] Nahmias J. S., Ramos R., Kraretzky M. (1995). Nasal CPAP in patients with obstructive sleep apnea: validation of a predictive equation. *Journal of Sleep Research*.

[66] Ito E., Tsuiki S., Namba K., Takise Y., Inoue Y. (2014). Upper airway anatomical balance contributes to optimal continuous positive airway pressure for Japanese patients with obstructive sleep apnea syndrome. *Journal of Clinical Sleep Medicine*.

[67] Suratt P. M., McTier R. F., Findley L. J., Pohl S. L., Wilhoit S. C. (1987). Changes in breathing and the pharynx after weight loss in obstructive sleep apnea. *Chest*.

[68] Malhotra A., Huang Y., Fogel R. B. (2002). The male predisposition to pharyngeal collapse: importance of airway length. *The American Journal of Respiratory and Critical Care Medicine*.

[69] Camacho M., Zaghi S., Certal V. (2015). Inferior turbinate classification system, grades 1 to 4: development and validation study. *The Laryngoscope*.

[70] Camacho M., Riaz M., Capasso R. (2015). The effect of nasal surgery on continuous positive airway pressure device use and therapeutic treatment pressures: a systematic review and meta-analysis. *Sleep*.

[71] Johns M. W. (1991). A new method for measuring daytime sleepiness: the Epworth sleepiness scale. *Sleep*.

